# Presenting Characteristics, Treatment, and Visual Outcomes in Streptococcal Compared to Non-Streptococcal Endophthalmitis

**DOI:** 10.7759/cureus.65974

**Published:** 2024-08-01

**Authors:** Richmond Woodward, Regina De Luna, Cason B Robbins, Henry L Feng, Jason E Stout, Sharon Fekrat

**Affiliations:** 1 Department of Ophthalmology, Duke University School of Medicine, Durham, USA; 2 Department of Comprehensive Ophthalmology, Advanced Eye Associates, San Jose, USA; 3 Department of Ophthalmology, Rush University Medical Center, Chicago, USA; 4 Department of Medicine, Duke University School of Medicine, Durham, USA

**Keywords:** non-streptococcal endophthalmitis, streptococcal endophthalmitis, bacterial endophthalmitis, endophthalmitis, infectious endophthalmitis

## Abstract

Purpose: Report the clinical findings, risk factors, treatment, and visual outcomes associated with *Streptococcus* endophthalmitis in comparison to culture-positive endophthalmitis associated with non-*Streptococcus* species.

Methods: A retrospective chart review of adults between 18 and 89 years of age diagnosed with exogenous culture-positive endophthalmitis between January 1, 2009, and January 1, 2018, at the Duke Eye Center (Durham, North Carolina) with at least six months of follow-up from time of initial diagnosis was conducted. Clinical data including patient demographics, ocular history, baseline corrected visual acuity (VA) prior to presentation, time to presentation, presenting exam findings, VA at presentation, presumed etiology of endophthalmitis, medical and surgical management, and VA at the six-month follow-up was extracted and statistically analyzed.

Results: Fifty-six eyes from 56 patients with culture-positive endophthalmitis were identified. Eyes with *Streptococcus* (n=18) had elevated intraocular pressure (IOP) at presentation (p=0.002), worse mean VA (Snellen) at presentation (20/14159 vs. 20/3098, p<0.001), and worse mean VA (Snellen) at six months (20/3475 vs. 20/235, p<0.001) compared to non-*Streptococcus* cases (n=38). Time to presentation (days) (median, IQR) was longer in eyes that underwent glaucoma surgery for both *Streptococcus *(2241 (836, 3709) vs. 3 (2, 31), p=0.003) and non-*Streptococcus *endophthalmitis (1236 (125, 3582) vs. 6 (4, 25), p<0.0001). There was no difference in VA at six months between *Streptococcus* and non-*Streptococcus* eyes based on treatment.

Conclusions: *Streptococci* are rare but important causes of exogenous endophthalmitis, and in our study, they were associated with worse visual outcomes than non-*Streptococci*. A history of any glaucoma surgery, even procedures performed years earlier, should be elicited when evaluating patients with ocular symptoms.

## Introduction

Endophthalmitis is a medical and ophthalmic emergency that requires suspicion on the part of clinicians to ensure prompt recognition, referral, and treatment. The etiology of endophthalmitis can be categorized as exogenous, when bacteria directly enter the eye via trauma, iatrogenic activity, or extension of corneal infection, or as endogenous, resulting from hematogenous spread of bacteria or fungi to the eye. Cases of culture-positive bacterial endophthalmitis are more commonly due to exogenous causes. Both categories of endophthalmitis can lead to severe visual loss.

Among bacterial causes of endophthalmitis, prior studies have suggested that endophthalmitis due to *Streptococcus pneumoniae* is clinically more severe, is associated with worse visual outcomes, and carries a higher risk of evisceration or enucleation compared to endophthalmitis caused by non-*Streptococcus* organisms [1]. Coagulase-negative *Staphylococci* and viridans streptococci, while generally less virulent than *S. pneumoniae*, cause most cases of post-intravitreal injection endophthalmitis and post-cataract surgery endophthalmitis [2,3]. Other non-*Streptococcus* bacteria implicated as the causative organism in exogenous endophthalmitis include *Haemophilus influenzae*, *Enterococcus*, and *Bacillus* species.

To the best of our knowledge, only one prior study has compared *Streptococcus* to non-*Streptococcus* culture-positive bacterial endophthalmitis. In a series of 47 cases of culture-positive bacterial endophthalmitis, endophthalmitis caused by *Streptococcus* species was associated with a worse final visual acuity (VA) compared to endophthalmitis caused by coagulase-negative *Staphylococcus*; other clinical features were not reported [4]. To gain additional insight into the clinical findings, risk factors, treatment, and visual outcomes associated with culture-positive *Streptococcus *endophthalmitis compared to endophthalmitis associated with non-*Streptococcus *species, we performed a retrospective study of cases of bacterial endophthalmitis at a large tertiary care center.

## Materials and methods

Study population

This retrospective, single-center study was approved by the Duke University Health System Institutional Review Board (Pro00091062). Patients between 18 and 89 years of age diagnosed with exogenous culture-positive endophthalmitis at the Duke Eye Center between January 1, 2009, and January 1, 2018, were identified using the Duke Enterprise Data Unified Content Explorer. Patients with exogenous culture-positive endophthalmitis and at least six months of follow-up from the initial presentation were included in the study. Specimens for microbial culture were obtained by aqueous tap, needle vitreous tap, or pars plana vitrectomy (PPV) with a mechanical vitreous biopsy. Culture-positive endophthalmitis was considered to be present if microbiological organisms were isolated on culture. 

Data collection

Data was collected from February 7, 2018, through February 6, 2019, and accessed for research purposes from February 7, 2019, through January 15, 2023. All patient information was anonymized for analysis and kept in a password-protected Excel file (Microsoft Corporation. 2021. Microsoft Excel. Redmond, WA) on a secure server. Data on patient demographics, ocular history, time to presentation, presenting ocular exam findings, presumed etiology of endophthalmitis, microbiologic culture yield, and medical and surgical management was extracted. Time to presentation was measured in days between an intraocular surgical procedure or occurrence of ocular trauma and the time an individual presented to ophthalmology for care. Corrected VA at baseline before endophthalmitis symptoms developed, at presentation of endophthalmitis, and at the six-month follow-up was extracted. Baseline, or pre-endophthalmitis VA, was collected from the most recent clinic visit prior to the development of endophthalmitis. 

Statistical analysis

Statistical analysis was performed using Stata (StataCorp. 2021. Stata Statistical Software: Release 17. College Station, TX: StataCorp LLC). Categorical variables were compared using Pearson’s Chi-squared test. Continuous variables for two sample groups normally distributed were compared using a two-tailed t-test and continuous variables for two sample groups not normally distributed were compared using Wilcoxon Rank Sum. Logistic regression analysis was used to identify associations between binary outcomes and continuous predictor variables. Snellen-equivalent VA was converted to the logarithm of the minimum angle of resolution (logMAR) for the purpose of statistical comparison. The logMAR equivalents of count fingers, hand motion, light perception (LP), and no light perception (NLP) were 2.3, 2.6, 2.9, and 3.2, respectively, as extrapolated from a previous study for measurement at one foot [5]. A p-value less than 0.05 was used in all analyses to define statistical significance. 

## Results

Clinical characteristics and bacterial species

Fifty-six patients with unilateral exogenous culture-positive endophthalmitis were identified. The mean age was 67 years (range: 30-85 years). Eighteen patients had positive ocular cultures for *Streptococcus* and 38 for non-*Streptococcus* species. There were no significant differences in age or sex between the two groups (Table 1).

**Table 1:** Demographic data and presenting ocular findings for culture-positive Streptococcus and non-Streptococcus endophthalmitis. IOP, intraocular pressure

Clinical characteristic	*Streptococcus* culture-positive (n=18)	Non-*Streptococcus* culture-positive (n=38)	P-value
Right eye, No. (%)	7 (39)	22 (58)	0.18
Female sex, No. (%)	8 (44)	26 (68)	0.086
Age (years), mean ± SD	65.4±17.6	68.5±13.6	0.47
Conjunctival hyperemia, No. (%)	16 (89)	24 (63)	0.047
Ocular pain, No. (%)	16 (89)	33 (87)	0.83
Elevated IOP (>21 mmHg), No. (%)	10 (56)	6 (16)	0.002
Hypotony (<9 mmHg), No. (%)	1 (6)	4 (11)	0.54
Hypopyon, No. (%)	13 (72)	24 (63)	0.50
Anterior chamber fibrin, No. (%)	17 (94)	26 (68)	0.031

Of the *Streptococcus* isolates, 9/18 (50%) were identified as *S. pneumoniae*, 8/18 (44%) were *Streptococcus viridans*, and 1/18 (6%) were *Streptococcus* *agalactiae*. Of the non-*Streptococcus* isolates, the most common isolates were 21/38 (55%) coagulase-negative *Staphylococci*, 5/38 (13%) *Cutibacterium acnes*, 5/38 (13%) *H. influenzae, *3/38 (8%), and *Bacillus* species 3/38 (8%) (Table 2).

**Table 2:** Bacterial species for culture-positive Streptococcus and non-Streptococcus endophthalmitis.

Bacterial species	Number (%) of *Streptococcus* species	Number (%) of non-*Streptococcus* species
Streptococcus pneumoniae	9 (50)	
Streptococcus viridans	8 (44)	
Streptococcus agalactiae	1 (6)	
Coagulase-negative *Staphylococci*		21 (55)
Cutibacterium acnes		5 (13)
Haemophilus influenzae		3 (8)
*Bacillus* species		3 (8)
*Enterococcus *species		2 (5)
Staphylococcus aureus		2 (5)
*Moraxella *species		1 (3)
*Pseudomonas* species		1 (3)

Causative etiologies

The etiologies for the 56 cases of culture-positive endophthalmitis included cataract surgery (15/56 (27%)), trabeculectomy (glaucoma filtration surgery) (12/56 (21%)), intravitreal injection (IVI) (8/56 (14%)), partial or full thickness corneal transplantation (7/56 (13%)), open globe injury (5/56 (9%)), glaucoma drainage device (GDD) insertion (5/56 (9%)), corneal ulcer (3/56 (5%)), and PPV (1/56 (2%)) (Table 3). Endophthalmitis was more likely to be culture-positive for *Streptococcus *species rather than non-*Streptococcus *species in the setting of open globe injury (OR 10.57, 95% CI 1.09-102.94, p=0.042) and trabeculectomy (OR 4.20, 95% CI 1.10-15.96, p=0.035).

**Table 3:** Distribution of causative etiologies of culture-positive endophthalmitis. a: none with IOFB; b: intravitreal injection; c: partial or full thickness; d: cataract surgery without intracameral antibiotics; e: PPV; f: GDD PPV, pars plana vitrectomy; GDD, glaucoma drainage device; IOFB, intraocular foreign body

	Open globe^a^	Glaucoma surgery		IVI^b^	Corneal transplant^c^	Cataract surgery^d^	Ulcer	PPV^e^
*Streptococcus* positive (N=18)	4	Trabeculectomy	7	1	2	0	2	0
		GDD^f^	2					
Non-*Streptococcus* positive (N=38)	1	Trabeculectomy	5	7	5	15	1	1
		GDD	3					

Presentation 

When compared to non-*Streptococcus* cases, *Streptococcus* endophthalmitis was significantly associated with initial exam findings of conjunctival hyperemia, elevated intraocular pressure (IOP), and increased anterior chamber fibrin (Table 1). There was no difference in baseline VA prior to presentation between patients with *Streptococcus* compared with non-*Streptococcus* endophthalmitis (p=0.72) (Table 4). Eyes with *Streptococcus* had worse mean VA (Snellen) at presentation compared to non-*Streptococcus* cases (20/14159 vs. 20/3098, p<0.001). At presentation, 13/18 (72%) of patients with *Streptococcus* had NLP or LP VA in the affected eye compared with 5/38 (13%) of patients with non-*Streptococcus* (p<0.001). Overall, there was no difference in time to presentation (days) (median, IQR) comparing patients with positive ocular cultures for *Streptococcus* to patients with positive ocular cultures for non-*Streptococcus* bacteria (Table 4). However, patients with a positive ocular culture for *Streptococcus* and a history of glaucoma surgery (trabeculectomy or GDD) had a longer time to presentation (days) (median, IQR) compared to patients with *Streptococcus* who did not undergo glaucoma surgery (2241 (836, 3709) vs. 3 (2, 31) p=0.003). Similarly, patients with a positive ocular culture for non-*Streptococcus* species and a history of glaucoma surgery (trabeculectomy or GDD) had a longer time to presentation (days) (median, IQR) compared to patients culture-positive for non-*Streptococcus* species who did not undergo glaucoma surgery (1236 (125, 3582) vs. 6 (4, 25), p<0.0001)). 

**Table 4:** VA for culture-positive Streptococcus and non-Streptococcus endophthalmitis. *Continuous variables for two sample groups normally distributed were compared using a two-tailed t-test and continuous variables for two sample groups not normally distributed were compared using Wilcoxon Rank Sum. Categorical variables were compared using Pearson’s Chi-squared test. A p-value less than 0.05 was used in all analyses to define statistical significance. VA, visual acuity; LP, light perception; NLP, no light perception

Characteristic	*Streptococcus* culture-positive (N = 18)	Non-*Streptococcus* culture-positive (N=38)	P-value*
Time to presentation (days) (median, IQR)	401 (2.5, 2149)	16 (4, 76)	0.25
VA pre-infection, mean (Snellen)	20/98	20/132	0.72
VA at presentation, mean (Snellen)	20/14159	20/3098	<0.001
LP or NLP at presentation, No. (%)	13 (72)	5 (13)	<0.001
VA at 6 months, mean (Snellen)	20/3475	20/235	<0.001
LP or NLP at 6 months, No. (%)	10 (56)	3 (8)	<0.001

Management and outcomes

Eyes with *Streptococcus* had worse mean VA (Snellen) at the six-month follow-up compared to non-*Streptococcus* cases (20/3475 vs. 20/235, P<0.001) (Table 4). At six months, 10/18 (56%) of patients with *Streptococcus* had LP or NLP VA compared to 3/38 (8%) of patients with non-*Streptococcus* endophthalmitis (p<0.001). 

All 18 patients with *Streptococcus* were treated with intravitreal injection of vancomycin on presentation, and 17/18 (94%) were also treated with intravitreal ceftazidime. Similarly, all non-*Streptococcus* patients were treated with intravitreal vancomycin on presentation, and 32/38 (84%) were also treated with intravitreal ceftazidime. 

Topical prednisolone acetate 1% was initiated on presentation in 14/18 (78%) patients with *Streptococcus* and of these patients, 7/14 (50%) also received an oral steroid. Among patients with *Streptococcus* endophthalmitis, there was no difference in change in VA from the time of presentation to the six-month follow-up for patients treated with topical steroids compared to patients treated with both topical and oral steroids (p=0.069). Three patients did not receive any topical or oral steroids and one patient received oral steroids only. 

## Discussion

An important finding from our study is that time to presentation was longer in patients with culture-positive *Streptococcus *endophthalmitis and patients with non-*Streptococcus* endophthalmitis that had undergone glaucoma surgery (trabeculectomy or GDD implant) compared to patients that had not undergone glaucoma surgery. Delayed presentation (> one month after surgery) of endophthalmitis following glaucoma surgery has been previously reported [6-9]. In a series of four eyes with culture-positive endophthalmitis following GDD implant, one eye developed endophthalmitis 24 months after GDD implant [6]. In a series of 53 eyes with delayed onset endophthalmitis following trabeculectomy, the mean time to onset of endophthalmitis was 64 months (SD: 46 months) [7]. Eyes presenting with endophthalmitis more than 10 years after trabeculectomy or GDD have been reported [8,9]. Our results for median time to presentation with endophthalmitis due to *Streptococcus *and non-*Streptococcus* species following glaucoma surgery are consistent with these published studies. 

To our knowledge, there are no published reports comparing the time to presentation of culture-positive endophthalmitis following glaucoma surgery with culture-positive endophthalmitis following other inciting etiologies. In contrast, our results demonstrated patients with endophthalmitis culture-positive for *Streptococcus *species and patients with endophthalmitis culture-positive for non-*Streptococcus* species have a longer time to presentation following glaucoma surgery compared to other inciting etiologies. These results suggest a history of any glaucoma surgery, even procedures performed years earlier, should be elicited when evaluating patients with ocular symptoms. While our study size did not allow for stratification of results for time to presentation by type of glaucoma surgery, e.g., trabeculectomy or GDD implant, reporting results as time to presentation following "glaucoma surgery" acknowledges patients may not recall the specific type of glaucoma surgery they underwent months or years later.

Delayed presentation of endophthalmitis following glaucoma surgery can be attributed to the inherent characteristics of the surgical procedure. During trabeculectomy, a defect is intentionally created in the eye wall. This defect allows aqueous fluid to filter into the subconjunctival space, forming an elevated blister known as a filtering bleb. In this area, the normal barrier provided by the eye wall is compromised or lost. It is hypothesized that after trabeculectomy, because only the conjunctiva separates ocular surface flora from the aqueous humor at the trabeculectomy site, causative organisms may migrate across the conjunctival epithelium into the anterior chamber of the eye and lead to endophthalmitis months or years after trabeculectomy surgery [10]. Delayed onset endophthalmitis associated with GDD implants may be due to bacterial migration from the surface of the eye following erosion and exposure of the drainage device itself [6]. The role of GDD erosion in the etiology of post-GDD implant endophthalmitis is shown by the rarity of endophthalmitis following GDD implant without tube shunt erosion [11].

Another unique feature of our study was comparing ocular exam characteristics at the time of presentation of endophthalmitis in patients culture-positive for *Streptococcus* with patients culture-positive for non-*Streptococcus* species. Our results demonstrated IOP at presentation was elevated in eyes with* Streptococcus* endophthalmitis compared to non-*Streptococcus* endophthalmitis. Previous reports of elevated IOP at the time of presentation of endophthalmitis have been limited to the setting of endophthalmitis following trabeculectomy [7,12]. Findings from our study expand beyond trabeculectomy-related endophthalmitis to show that IOP at the time of presentation of culture-positive endophthalmitis due to *Streptococcus *species was associated with a range of inciting etiologies. 

Elevated IOP at the time of presentation of endophthalmitis may be due to inflammation of the trabecular meshwork, or inflammatory debris trapped in the trabecular meshwork, similar to the proposed mechanism of elevated IOP in inflammatory ocular hypertension syndrome [13]. A greater inflammatory response triggered by streptococcal exotoxins and enzymes, and the subsequent effect of this inflammation on the trabecular meshwork, may explain our results demonstrating elevated IOP at the time of presentation of endophthalmitis culture-positive for *Streptococcus* species [14]. 

Endophthalmitis was more likely to be culture-positive for *Streptococcus *species compared to non-*Streptococcus* species following open globe injury in our study. Most cases of post-traumatic endophthalmitis are caused by bacteria present in the environment. Risk factors for developing post-traumatic endophthalmitis include delayed globe closure, trauma in a rural setting, wound contamination, presence of an intraocular foreign body (IOFB), and lens capsule disruption [15]. In a series of 565 bacterial isolates from 581 eyes with endophthalmitis following open globe injury, the most common isolate was *Bacillus* species, followed by *S. pneumonia* and coagulase-negative *Staphylococci* [16]. *Bacillus *species are commonly found in cases of post-traumatic endophthalmitis with IOFB or soil contamination [17]. An explanation for our finding of greater odds of isolating *Streptococcus* species compared to non-S*treptococcus* species from patients with culture-positive endophthalmitis following open globe injury is that no eyes with open globe injury in our study had an IOFB. 

Endophthalmitis was also more likely to be culture-positive for *Streptococcus* rather than non-*Streptococcus* species in the setting of trabeculectomy in our study. This is consistent with a previously reported series of 38 eyes with endophthalmitis caused by *Streptococcus* species, where the most common clinical setting was post-glaucoma surgery, in particular trabeculectomy [18]. Similarly, in a series of 39 eyes that developed culture-positive endophthalmitis after trabeculectomy, the most frequently isolated organisms were *Streptococcus *species [19]. A possible explanation for our results finding greater odds of isolating *Streptococcus *species compared to non-*Streptococcus* species in patients with endophthalmitis following trabeculectomy is that, unlike *Streptococci*, *Staphylococci* do not produce exotoxins and may not have the ability to penetrate intact conjunctiva [20]. 

Endophthalmitis culture-positive for *Streptococcus* species was associated with poorer VA at presentation and at six months compared to endophthalmitis culture-positive for non-*Streptococcus* species in our study. Greater than 50% of patients with *Streptococcus *endophthalmitis in our study had poor VA (NLP or LP) at the time of presentation and at six months. These results are consistent with other studies showing poor visual outcomes associated with culture-positive *Streptococcus* endophthalmitis despite prompt and appropriate treatment. In a series of 63 eyes treated for endophthalmitis due to *Streptococcus* species, the mean Snellen VA at presentation was 20/4900 (equivalent to hand motion VA), improving to 20/2900 at follow-up [21]. The severe impact of *S. pneumoniae* endophthalmitis on VA outcome was demonstrated in a study of 38 eyes treated for *S. pneumoniae* endophthalmitis where the final VA was NLP in 30/38 (79%) [1]. 

The reasons why patients with culture-positive *Streptococcus* endophthalmitis have poor visual outcomes compared to other etiologic bacteria are not fully understood. Studies of a capsule-deficient strain of* S. pneumoniae* in rabbits showed endophthalmitis caused by the encapsulated strain is more damaging to retinal function than the capsule-deficient strain, suggesting the capsule is an important virulence factor in endophthalmitis [22]. Additionally, the *S. pneumoniae* toxin pneumolysin creates a virulence factor that causes damage as a pore-forming toxin and may mediate inflammation through activation of the classical complement pathway [23]. However, a study of streptococcal-specific virulence factors in eyes with culture-positive *Streptococcus* endophthalmitis found no association between the presence of pneumolysin, autolysin, and hyaluronidase and visual outcome or rate of enucleation [18]. While antibiotics kill intraocular organisms, they do not directly affect the toxins or enzymes that mediate inflammation and adversely affect retinal function [24]. These findings suggest any number of factors related to the characteristics of *Streptococci*, either individually or in combination, may contribute to the poor VA outcomes associated with endophthalmitis due to *Streptococcus* species despite treatment.

Limitations

Limitations of the current study include its retrospective design and relatively small number of patients. However, the frequency of cases in this study, 56 eyes over a nine-year period, fits with another published series of culture-positive endophthalmitis from a single institution where 47 cases of culture-positive bacterial endophthalmitis over 12 years were reported [4]. Regarding management, our study did not attempt to analyze antibiotic susceptibility patterns due to limited susceptibility data. However, as a single-center study, the management of cases was largely homogenous, and patients did not come from a wide geographic area with potentially different patterns of antibiotic resistance, minimizing the impact on outcomes.

## Conclusions

*Streptococci* are rare but important causes of exogenous endophthalmitis, and in our study, they were associated with worse visual outcomes than non-*Streptococci*. Endophthalmitis was more likely to be culture-positive for *Streptococcus* species rather than non-*Streptococcus* species in the setting of open globe injury and trabeculectomy (glaucoma filtration surgery). Time to presentation was longer in patients with culture-positive *Streptococcus* endophthalmitis and non-*Streptococcus* endophthalmitis that had undergone glaucoma surgery (trabeculectomy or GDD implant) compared to patients with culture-positive endophthalmitis following other inciting etiologies. A history of any glaucoma surgery, even procedures performed years earlier, should be elicited when evaluating patients with ocular symptoms. While our study had limited power, we did not see any effect of the use of adjunctive corticosteroids. Prompt antimicrobial therapy and intervention are key to optimizing outcomes among patients with culture-positive bacterial exogenous endophthalmitis.
